# Serum Induces the Subunit-Specific Activation of NF-κB in Proliferating Human Cardiac Stem Cells

**DOI:** 10.3390/ijms25073593

**Published:** 2024-03-22

**Authors:** Kazuko E. Schmidt, Anna L. Höving, Katja Nowak, Nike an Mey, Sina Kiani Zahrani, Britta Nemeita, Lena Riedel, Agnes Majewski, Barbara Kaltschmidt, Cornelius Knabbe, Christian Kaltschmidt

**Affiliations:** 1 Department of Cell Biology, Faculty of Biology, University of Bielefeld, 33615 Bielefeld, Germanynike.mey@uni-bielefeld.de (N.a.M.); sina.kiani@uni-bielefeld.de (S.K.Z.); britta.nemeita@uni-bielefeld.de (B.N.);; 2 Institute for Laboratory and Transfusion Medicine, Heart and Diabetes Centre NRW, Ruhr-University Bochum, 32545 Bad Oeynhausen, Germany; cknabbe@hdz-nrw.de; 3 Medical Faculty Ostwestfalen-Lippe, University of Bielefeld, 33615 Bielefeld, Germany; 4 AG Molecular Neurobiology, Faculty of Biology, University of Bielefeld, 33615 Bielefeld, Germany; barbara.kaltschmidt@uni-bielefeld.de

**Keywords:** human cardiac stem cells, proliferation, human blood serum, NF-κB, PDTC, RNA-Seq

## Abstract

Cardiovascular diseases (CVDs) are often linked to ageing and are the major cause of death worldwide. The declined proliferation of adult stem cells in the heart often impedes its regenerative potential. Thus, an investigation of the proliferative potential of adult human cardiac stem cells (hCSCs) might be of great interest for improving cell-based treatments of cardiovascular diseases. The application of human blood serum was already shown to enhance hCSC proliferation and reduce senescence. Here, the underlying signalling pathways of serum-mediated hCSC proliferation were studied. We are the first to demonstrate the involvement of the transcription factor NF-κB in the serum-mediated proliferative response of hCSCs by utilizing the NF-κB inhibitor pyrrolidine dithiocarbamate (PDTC). RNA-Sequencing (RNA-Seq) revealed *ATF6B*, *COX5B*, and *TNFRSF14* as potential targets of NF-κB that are involved in serum-induced hCSC proliferation.

## 1. Introduction

The physiological decline and the accompanied decreasing regenerative capacity of the body, which occur during our lifetimes, are the major causes of age-related diseases. In 2013, López Otín et al. described nine hallmarks of ageing comprising cellular senescence and stem cell exhaustion [1]. The cellular viability and proliferation of adult stem cell populations is crucial for maintaining healthy tissues and organs as they mainly contribute to the regeneration of the body [1]. Cardiovascular diseases (CVDs) are strongly linked to an ageing phenotype and are the leading cause of death worldwide, which makes the investigation of cardiac stem cell populations of great interest for developing new treatments that enhance the clinical outcome of CVDs [2,3]. The adult heart has long been considered as an organ with a low regenerative capacity that lacks endogenous stem cell populations, until cardiac stem cells (CSCs) were discovered in the mouse and human heart. The described CSC populations were characterized by the expression of Sca1+/CD105+/CD31+ and the stem cell factor receptor kinase c-KIT [4,5,6,7]. However, a range of c-KIT-negative CSC populations have also been described [8]. Interestingly, these cells have been reported to have proliferative and regenerative capacity. Particularly, CSCs were shown to differentiate into cardiomyocytes. In previous studies, we successfully isolated and characterized a human cardiac stem cell (hCSC) population from human left atrial appendages (LAAs) [7,9]. This population was c-KIT-negative and expressed the stem cell markers Nestin and S100, as well as Sca1, CD105, and CD31. Notably, we could demonstrate the ability of this hCSC population to form cardiospheres after clonal growth and to differentiate into cardiomyocyte-like cells in vitro [7]. The described population can potentially contribute to cardiac regeneration. Therefore, analysing the molecular basis of hCSC proliferation is a promising target for developing new strategies for CVD treatment.

Whole blood and blood serum from young donors have been shown to have rejuvenating effects on old tissues and organs in the murine system, but the translation to the human system remains challenging [10,11,12]. Besides rejuvenation, several beneficial effects of human blood serum have been reported by in vivo and in vitro studies and have been successfully approved in medical applications, such as serum therapy, which is effective in patients recently infected with SARS-CoV-2 [13]. In the clinical context, human blood plasma is already used for blood plasma transfusion or as treatment for burns, shocks, trauma, and infection. During the therapy of autoimmune diseases and haemophilia, human blood plasma contributes to an improved clinical outcome for the patient [14]. Therefore, further medical studies utilizing human blood plasma and serum from young donors are currently subjected to (pre-)clinical trials [8].

We successfully demonstrated an activating effect of human blood plasma and serum on hCSCs in previous in vitro studies [7,9,15]. In detail, both human blood plasma and serum were shown to significantly increase hCSC proliferation and cell viability, while serum application simultaneously reduced senescence in hCSCs. RNA-Seq analysis indicated the p38-MAPK signalling pathway as a major mediator of these effects. Inhibition of p38-MAPK upon serum or plasma treatment reduced the reported proliferation and protection from senescence in hCSCs, thereby confirming p38-MAPK signalling as a crucial regulator of the beneficial serum effects [7]. Besides proliferation, we could show that hCSC migration was induced by the application of human blood serum and was diminished by the inhibition of p38-MAPK signalling. Further analysis revealed heat shock protein 27 (Hsp27) as a direct target of serum-induced p38-MAPK signalling [16]. To gain deeper insights on the molecular mechanisms of the serum-induced effects, subsequent studies considering upstream and downstream signalling are necessary. In this context, we recently demonstrated the TGFβRI/II-SMAD2/3 pathway as a mediator of serum-induced hCSC proliferation. We further identified TGFβ1 as a proliferative and senescence-protective component contained in serum. Subsequent RNA-Seq analysis indicated that the TGFβ receptor type II (TGFβRII) regulates non-canonical pathways, which contribute to the prevention of hyperproliferation via Ras signalling [15]. Interestingly, previous transcriptomic analyses also showed that tumour necrosis factor alpha (TNF) signalling is upregulated in serum-treated hCSCs [7]. The NF-κB signalling pathway is known to regulate cell survival, proliferation, as well as the immune and inflammatory responses. However, the role of NF-κB regarding cardiac cells and tissues or serum-mediated proliferation has not been extensively studied so far. The NF-κB transcription factor family comprises five subunit proteins sharing a REL homology domain (RHD) at the N-terminus, which is significant for dimerization, nuclear translocation, and DNA binding. Namely, the subunits RelA (p65), RelB, and c-Rel share a transactivation domain (TAD), while the subunits p50 and p52 do not contain a TAD. The different NF-κB subunits are retained in the cytoplasm as heterodimers consisting of the different subunits by the inhibitor IκB. Upon activation of the signalling cascade, the inhibitor kinase complex (IKK) will be phosphorylated, which in turn phosphorylates the IκB and results in the release of the NF-κB heterodimer, allowing nuclear translocation. Subsequently, NF-κB target genes are activated after binding of the transcription factor [17].

In this study, we demonstrate the crucial role of NF-κB signalling in the serum-mediated proliferative response of hCSCs by cellular assays utilizing the NF-κB inhibitor pyrrolidine dithiocarbamate (PDTC). The application of PDTC resulted in a significant decline of hCSC proliferation upon serum treatment. The activation of NF-κB signalling by human blood serum was confirmed by immunocytochemical analysis of the subunits containing a TAD, namely RelA, RelB, and c-Rel. Additionally, the inhibitory effect of PDTC on nuclear translocation of these subunits was confirmed with immunocytochemistry (ICC). To unravel the molecular basis of the observed effects, RNA-Seq and subsequent promotor analysis were performed, revealing potential NF-κB targets activated by human blood serum.

## 2. Results

### 2.1. Inhibition of NF-κB Signalling Reduces Serum-Induced Cell Proliferation of hCSCs

**Figure 1:**
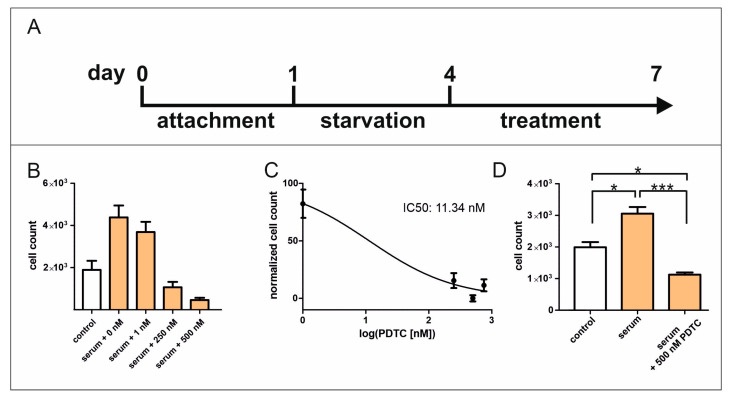
PDTC strongly reduces the proliferation of serum-treated hCSCs. (**A**) After starvation, hCSCs were treated with human blood serum and the NF-κB inhibitor PDTC. (**B**) Increasing amounts of PDTC strongly reduced the proliferation of hCSCs after serum treatment with a maximum effect at 500 nM. (**C**) The IC_50_ value of PDTC is 11.34 nM. (**D**) The application of 500 nM PDTC significantly reduced the proliferative effect of human blood serum on hCSCs below the cell count of untreated cells, therefore indicating cell death. (hCSC donor *n* = 2, human blood serum donors *n* = 6; technical replicates *n* = 3, Mann–Whitney U, * *p* < 0.05, *** *p* < 0.001).

Understanding the molecular mechanisms of hCSC proliferation after serum treatment is still of great interest for enhancing the potential treatments of CVDs. In previous studies, we successfully verified the significant participation of the p38-MAPK/HSP27 axis and TGFβRI/II-SMAD2/3 signalling in the context of serum-mediated hCSC proliferation [7,15,16]. In addition, the enrichment of TNF signalling was demonstrated after serum treatment in hCSCs [7]. The subsequent investigation of the NF-κB signalling pathway, which is strongly connected to MAPK and TNF signalling, might be crucial for resolving the mechanisms of hCSC proliferation induced by human blood serum. Pyrrolidine dithiocarbamate (PDTC) is a NF-κB inhibitor which acts by preventing the nuclear translocation of NF-κB heterodimers to the nucleus [18]. After attachment hCSCs were cultivated in serum-deprived starvation medium for 72 hours followed by treatment for additional 72 h (Figure 1A). Here, increasing amounts of PDTC were applied to serum-treated hCSCs, resulting in a proliferation-reducing effect after the addition of 1 nM PDTC. The application of 250 nM resulted in a cell count lower than in untreated cells, while the maximum effect was shown after the application of 500 nM PDTC (Figure 1B), resulting in an IC_50_ value of 11.34 nM (Figure 1C). The reported proliferative effect of human blood serum was significantly diminished by PDTC, resulting in a cell count lower than for untreated hCSCs (Figure 1D). Light microscopy images revealed morphological changes towards granular shape, therefore indicating cell death (Figure S1), which is in accordance with the results of the proliferation assay.

### 2.2. Human Blood Serum Induces NF-κB Subunit Translocation in hCSCs

**Figure 2:**
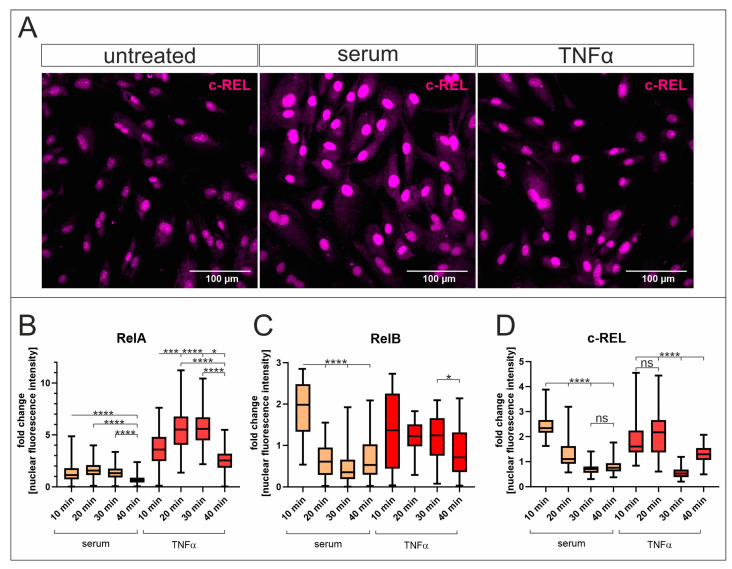
Human blood serum induces NF-κB-activation by nuclear translocation of the subunits RelA, c-Rel, and RelB. (**A**) Subsequent to confocal laser scanning microscopy, the nuclear fluorescence intensity was quantified and normalized to untreated cells to determine the subunit translocation from cytosol to the nucleus. Exemplary pictures of c-Rel are shown. (**B**–**D**) All three NF-κB subunits containing a transactivation domain showed the highest degree of nuclear translocation after 10 to 20 min of treatment in hCSCs, while the maximum effect of serum was visible for c-Rel translocation to the nucleus. (**C**,**D**) In addition, RelB and c-Rel show a higher activation in response to serum than to TNFα. (hCSC *n* = 1, serum from male donors *n* = 6 (pooled), Kruskal-Wallis test, * *p* < 0.05; *** *p* < 0.0005; **** *p* < 0.0001, ns = not significant).

**Figure 3:**
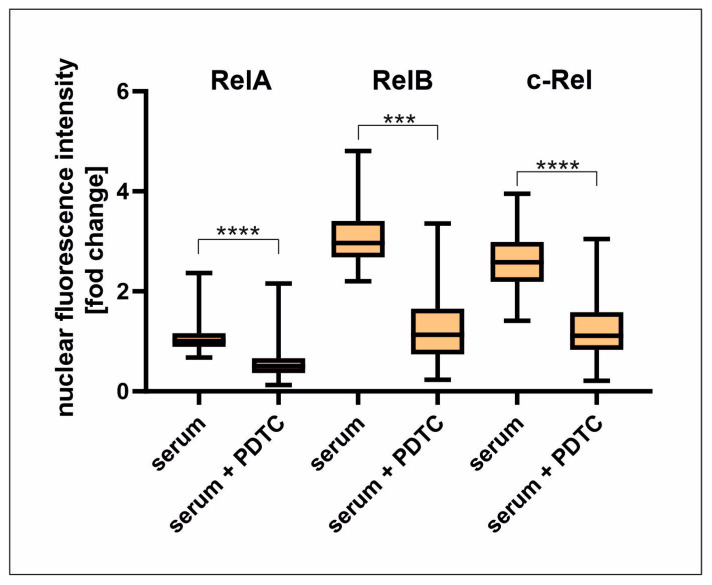
PDTC significantly reduces the nuclear translocation of NF-κB subunits upon serum treatment. Nuclear fluorescence intensity was quantified and normalized to untreated cells to determine the subunit translocation from cytosol to the nucleus. The induction of the subunit activation of RelA, RelB, and c-Rel was significantly reduced via PDTC after treatment with human blood serum. (hCSC *n* = 1, serum from male donors *n* = 6 (pooled), serum from female donors *n* = 6 (pooled), Kruskal-Wallis test, *** *p* < 0.0005; **** *p* < 0.0001).

Since NF-κB signalling seems to be important for the serum-mediated proliferation of hCSCs, we further examined its molecular effects. To determine the induction of NF-κB by human blood serum, immunocytochemical analysis was performed for the NF-κB subunits containing a transactivation domain RelA, RelB, and c-Rel. For RelA, the highest nuclear fluorescence intensity was visible after 20–30 min of TNFα treatment. In contrast, human blood serum induced RelA activation after a treatment duration of 10 min, but to a much lesser extent than after treatment with TNFα (Figure 2A). Serum treatment significantly induced the highest nuclear translocation of RelB after 10 min, while TNFα had a similar effect but was not considered significant after statistical analysis (Figure 2C). Likewise, the NF-κB subunit c-Rel was activated by TNFα after 10–20 min and by serum after 10 min (Figure 2D). Interestingly, human blood serum had a higher inductive effect than TNFα on the subunits RelB and c-Rel (Figure 2B,C). The inductive effect of human blood serum on RelA, RelB, and c-Rel nuclear translocation was successfully diminished via PDTC (Figure 3 and Figure S2).

### 2.3. RNA-Sequencing Reveals Upregulation of Cellular Ion Response GO-Terms and Downregulation of Cell Cycle KEGG Pathways in Serum and PDTC-Treated hCSCs

**Figure 4:**
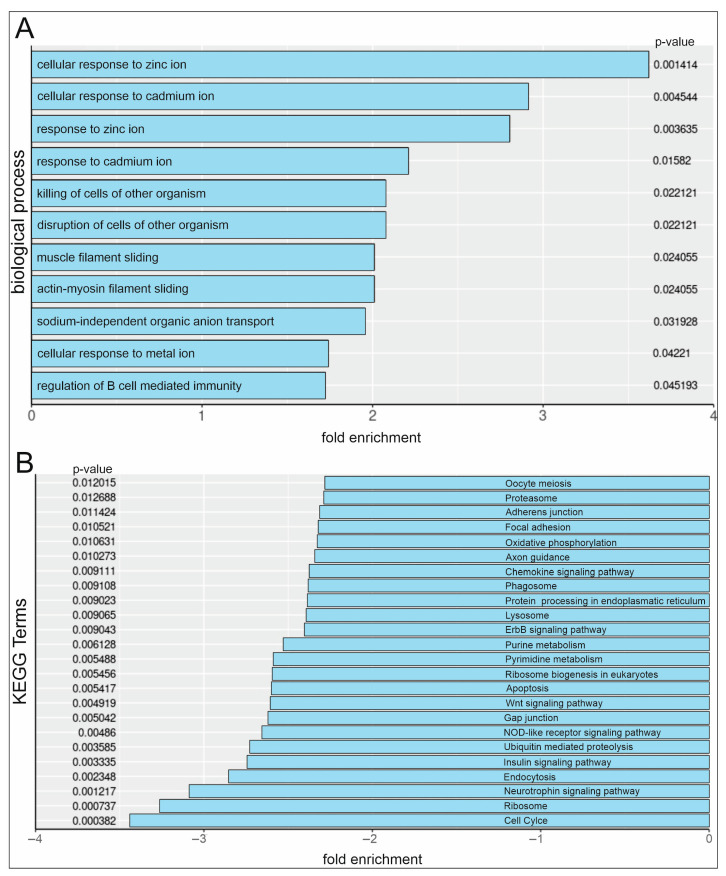
Pathway analysis of hCSCs after serum and PDTC treatment. (**A**) GO-Term analysis reveals the upregulation of cellular responses to metal ions in serum and PDTC-treated hCSCs. (**B**) KEGG pathway analysis shows downregulation of cell cycle in serum and PDTC-treated hCSCs.

Serum or serum and NF-κB inhibitor PDTC-treated hCSCs were analysed via RNA-Seq. Furthermore, pathway analysis after serum and PDTC treatment in hCSCs shows a significant enrichment of Gene Ontology (GO)-Terms in the category ‘biological process’ that are strongly related to cellular responses to several metal ions. In detail, the terms ‘cellular response to zinc ion’, ‘cellular response to cadmium ion’, ‘response to zinc ion’, ‘response to cadmium ion’, and ‘cellular response to metal ion’ were enriched (Figure 4A). Kyoto Encyclopaedia (KEGG) pathway analysis revealed a significant downregulation of the ‘cell cycle’ pathway (Figure 4B), which is in accordance with the observed reduction in proliferation.

### 2.4. RNA-Sequencing Reveals New NF-κB Target Genes in Serum-Treated hCSCs

**Figure 5:**
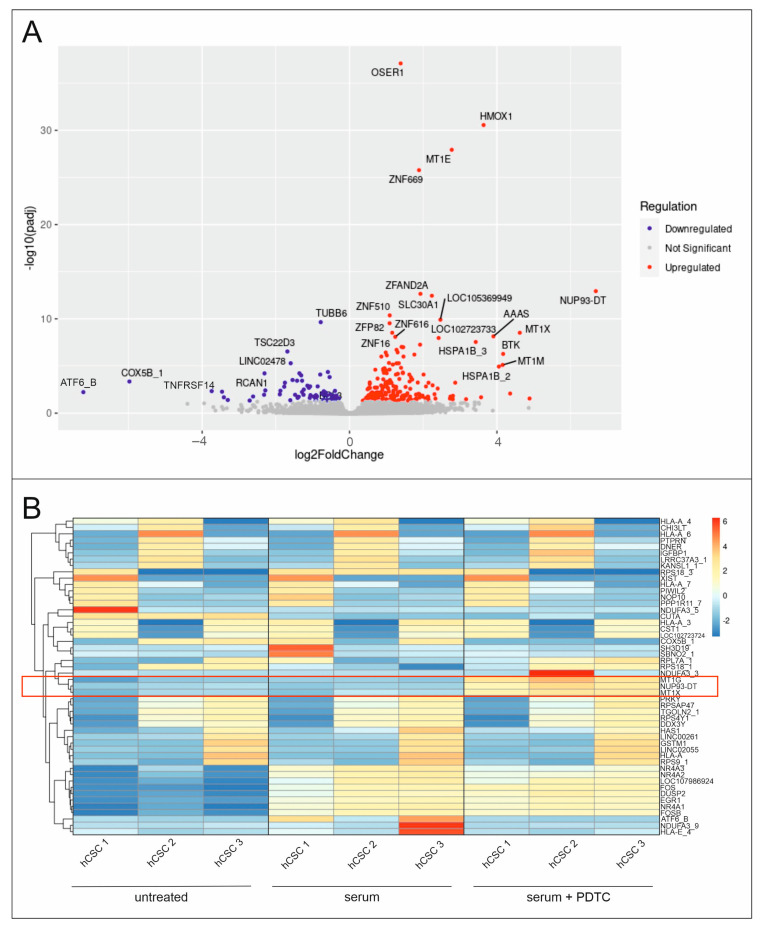
Differential gene expression analysis of hCSCs after treatment with the NF-κB inhibitor PDTC and human blood serum. (**A**) Vulcano plot reveals significant upregulation of 212 genes and significant downregulation of 66 genes. (**B**) Heatmap shows differential expression of *MT* genes after serum and PDTC treatment, highlighted by red box.

**Figure 6:**
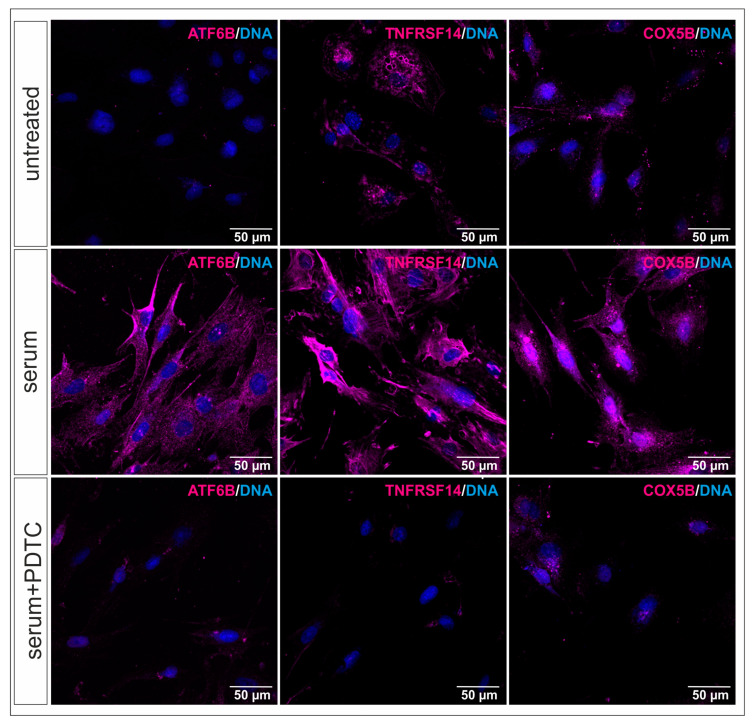
ATF6B, TNFRSF14, and COX5B are involved in the NF-κB-regulated response to serum in hCSCs. Immunocytochemical staining of hCSCs shows induction of ATF6B, COX5B, and TNFRSF14 protein expression upon serum treatment compared to untreated cells. This effect is reduced in serum and NF-κB inhibitor PDTC-treated cells compared to serum-treated cells, thereby indicating potential gene expression regulation of *ATF6B*, *COX5B*, and *TNFRSF14* via NF-κB.

RNA-Seq results were investigated in detail regarding the involved pathways. Therefore, hCSCs were treated with human blood serum, and gene expression was compared to hCSCs treated with human blood serum and the NF-κB inhibitor PDTC. The significant upregulation of 212 genes and downregulation of 66 genes was revealed via differential gene expression analysis (Figure 5A,B). Notably, genes comprising several subtypes of methalothionines (MT) and zinc finger (ZNF) were significantly upregulated. Genes encoding for *ATF6B*, *COX5B*, as well as *TNFRSF14* were highly downregulated after PDTC treatment, with fold changes ranging from −3.46 to −7.22. Promotor analysis suggests NF-κB binding at multiple putative binding sites of these genes (Table 1). Immunocytochemical staining of serum-treated hCSCs shows induced ATF6B, COX5B, and TNFRSF14 protein expression compared to untreated cells. This effect was reduced using a simultaneous treatment with serum and PDTC. These results indicate the involvement of all three targets in the serum response of hCSCs and demonstrate a potential regulation via NF-κB (Figure 6).

## 3. Discussion

Age-related diseases are mainly caused by the declining regenerative potential of the body during our lifetimes [1]. Especially, CVDs are strongly linked to the ageing phenotype and are the major cause of death worldwide [19,20]. As the heart has long been considered as an organ with low regenerative potential, the investigation of cardiac cell populations, particularly CSCs, is of great interest for improving and developing clinical approaches. Adult stem cells mainly contribute to the regeneration of tissues and organs, underlining stem cell exhaustion as major hallmark of ageing and making the investigation of stem cell proliferation, as a marker for stem cell fitness, crucial [1,21,22]. So far, CSC populations have been isolated from the murine and human heart and identified by the expression of the markers Sca1+/CD105+/CD31+ and partially the stem cell factor receptor kinase c-KIT [4,5,6]. Several studies already demonstrated a CSC-based improvement in cardiac regeneration, and clinical trials are currently pending [23]. We successfully obtained hCSCs from human LAAs expressing the markers Nestin and S100, as well as the cardiac progenitor markers Sca1, CD105, and CD31 in previous studies [7]. This population is c-KIT-negative and is able to form cardiospheres after clonal growth and differentiate into cardiomyocyte-like cells in vitro [7]. Based on their stem-cell like characteristics and differentiation potential, the described cardiac stem cell population harbours great potential for contributing to enhanced cardiac regeneration. In this context, blood plasma and serum from young donors have been shown to rejuvenate old tissues and organs and are tested in several clinical trials [8]. Particularly, we could show an inductive effect of human blood plasma and serum on hCSC proliferation and protection from senescence, whereby the p38-MAPK/HSP27 axis is identified as a crucial mediator of this effect [7]. Previous RNA-Seq analysis revealed the upregulation of p38-MAPK and TNF signalling in hCSCs after treatment with human blood serum from young donors. Further, we demonstrated the involvement of TGFβRI/II and SMAD2/3 signalling in serum-mediated hCSC proliferation [15]. Notably, a crosstalk of TGFβ and NF-κB has been reported via TGFβ-activated kinase 1 (TAK1) [24]. Based on these findings, the investigation of NF-κB signalling as a downstream pathway was focused on. The transcription factor NF-κB is known to be involved in several cellular processes, such as survival, immune and inflammatory responses, and proliferation [17,25,26]. To this day, the role of NF-κB has not been studied in detail regarding cardiac regeneration nor serum-mediated cellular responses on a molecular level. In this regard, the NF-κB-activating molecule TNFα was applied in proliferation assays, but an inductive effect on hCSC proliferation could not be assigned (Figure S3). Further, the NF-κB inhibitor PDTC was utilized in proliferation assays to elucidate the role of this signalling pathway in the serum-mediated effects of hCSCs. We could show that PDTC was able to reduce serum-induced hCSC proliferation in nanomolar concentrations with an IC_50_ value of 11.34 nM and a maximum effect of 500 nM. The simultaneous application of human blood serum and PDTC reduced the total cell count compared to untreated cells, indicating cell death. This was confirmed via light microscopy, which revealed granular-shaped, dead cells. The apoptotic effects of PDTC have been shown for human acute myelogenous leukemic cells [27], but the effect was shown to depend on cell type and the presence of Cu^2+^ and Zn^2+^ [28]. Oppositely, the inhibition via PDTC has been reported to prevent NF-κB-mediated apoptosis in HL-60 cells and thymocytes [29]. We suggest that NF-κB is not only involved in serum-mediated proliferation, but it is also essential for hCSC survival at a basal level and further suggests that PDTC has a cytotoxic effect. Our results are in accordance with studies reporting an inductive effects of PDTC in serum-exposed conditions of osteoblasts [30] and a linked anti-apoptotic function of NF-κB, which has been shown to be inactivated via PDTC application [31,32]. Additionally, PDTC has been shown to reduce the proliferation of gastric mucosal epithelial cells [33]. PDTC is currently subjected as a potential agent in clinical research; therefore, its effects on healthy stem cell populations necessary for regeneration have to be considered to prevent strong side effects [34,35]. Nevertheless, beneficial effects, such as a reduction of inflammatory responses, have been reported, and the drug was qualified to be valuable after toxicity analysis in mice and rats [36,37,38].

After identifying NF-κB as a mediator of serum-induced hCSC proliferation, the detailed mechanisms of activation were examined via the analysis of the nuclear translocations of the subunits containing a TAD, namely RelA, RelB, and c-Rel. Human blood serum successfully induced the nuclear translocation of RelA after 10 min but to a much lesser extent than after application of TNFα. The highest signal of the subunits RelB and c-Rel was detected in the nucleus after a 10 min serum application, while the extent was higher than after treatment with TNFα. Human blood serum showed an effect on the nuclear translocation of all three subunits, while the effect was higher for RelB and c-Rel, potentially indicating a subunit-specific mechanism. Interestingly, a subunit-specific role of NF-κB has also been reported in cancer [39]. Overall, a direct inductive effect of human blood serum on NF-κB subunit activation in hCSCs was confirmed. This effect has also been reported for serum from closed head injury (CHI) mouse serum on microglia in the context of neuronal damage and repair [40], and for human serum derived from patients with septic shocks in human cardiac myocytes [41]. In this study, we further demonstrated the inhibiting effect of PDTC on nuclear NF-κB subunit translocation in hCSCs. The mechanism underlying this effect is based on the prevention of the release of the inhibitory subunit IκB [18]. This subunit is responsible for holding the NF-κB subunit heterodimers in the cytoplasm until activation [17]. By preventing the release of IκB, the overall NF-κB transcription factor activity is inhibited [18]. To further elucidate the mechanisms contributing to the reductive effect of PDTC in serum-mediated hCSC proliferation, RNA-Seq was performed. In coherence with the observed reducing effects of PDTC on serum-mediated hCSC proliferation, the KEGG pathway “cell cycle” was downregulated 3-fold, while, strikingly, “apoptosis” was also downregulated 2-fold. Interestingly, several GO-Terms related to biological processes comprising cellular responses to two divalent cations with similar ionic radii, namely a zinc ion (0.74 Å (+2)) and cadmium (0.95 Å (+2)), as well as to metal ions, were significantly enriched. Cadmium (Cd) is a chemical element belonging to the Zn group and often occurs together with Zn [42]. It is known to induce MT expression for detoxification from Cd [43]. We suggest that the metal chelating properties of PDTC increased the intracellular zinc and cadmium levels. The capability of PDTC to introduce external metal to the intracellular regions has already been reported for zinc and copper [44,45]. Further differential gene expression analysis underlines this hypothesis as several genes encoding for detoxifying metallothioneins and zinc finger proteins were differentially expressed. In detail, we show the significant upregulation of 212 genes after treatment with human blood serum and PDTC compared to treatment with serum alone. These genes comprised several sequences encoding for metallothionines (MT), such as *MT1E*, *MT1X*, and *MT1M.* Similarly, to PDTC, the metallothionein protein has been reported to possess an iron-chelating function [43]. Further, MT has been identified as a modulator of NF-κB activity, which potentially indicates the upregulation of those genes as an intracellular rescue response to PDTC-induced NF-κB inhibition [43]. Additionally, genes encoding for zinc finger proteins (ZNFs), such as *ZNF669*, *ZNF510*, and *ZNF16*, were differentially expressed. ZNFs have been demonstrated to modulate the NF-κB signalling pathway by either repressing or activating transcription factor activity [43,46], but they are also capable of binding metals [47]. Potentially, the here-observed upregulation of genes encoding zinc and iron-chelating proteins is a consequence of increased intracellular zinc and iron to toxic levels, which were generated by the chelating actions of PDTC. Interestingly, for cerebral endothelial cells, it was reported that increased concentrations of zinc ions obtained from extracellular sources are necessary for PDTC-induced cell death [48]. The presence of Zn^2+^ has been crucial for the antiviral effects of PDTC, which were achieved by the transport of zinc ions to infected cells [44]. In contrast, intracellular zinc has also been described to prevent apoptosis and acts as a cofactor for essential cellular proteins comprising transcription factors and enzymes, while excessive amounts of zinc were shown to be cytotoxic [49,50]. In addition, PDTC has been demonstrated to have pro-oxidant properties that showed toxic effects in thymocytes. In detail, by introducing external copper to the cells, PDTC has been inducing oxidative stress, resulting in apoptosis [45]. In this context, our results suggest a PDTC-mediated cellular Zn^2+^ increase to toxic levels, which cannot be diminished due to the inhibited protection of NF-κB.

By investigating the 66 significantly downregulated genes after serum treatment and NF-κB inhibition, potential targets of the transcription factor were revealed. Notably, the expression of the *Activating transcription factor beta* (*ATF6B*) gene was reduced 7-fold, while promotor analysis confirmed three putative binding sites of NF-κB to the *ATF6B* gene. Compared to untreated cells, immunocytochemical staining showed induced ATF6B, COX5B, and TNFRSF14 protein expression in hCSCs, therefore demonstrating involvement in the serum response of hCSCs. Simultaneous treatment with serum and PDTC reduced protein expression compared to serum-treated hCSCs, thereby confirming a potential regulation via NF-κB. The members of the ATF family are known to be involved in several cellular processes, such as proliferation and apoptosis or cell redox homeostasis [51]. In this context, we very recently showed the significant upregulation of ATF3 and ATF6B mRNA expression in the response to serum in hCSCs and identified TGFβRI/II and SMAD2/3 signalling as crucial mediators of serum-induced proliferation [15]. Interestingly, the members of the ATF family were reported to regulate TGFβ signalling in several cell types [52,53,54,55]. In coherence with our previous studies [7], ATF6B is also known to be involved in the TNF signalling pathway as the downstream target of the p38-MAPK signalling cascade [56,57]. Additionally, ATFa, ATF2, and ATF3 have been previously identified to interact with NF-κB [58,59]. Notably, ATF4 and ATF6 were reported to regulate cell survival together with NF-κB in signal transduction pathways correlated with unfolded protein responses by endoplasmic reticulum stress [60]. Further studies suggested a protective effect of ATF6 and a NF-κB signalling reduction induced by Zn in the oxidative stress response to arsenic [61]. Moreover, the expression of *Cytochrome oxidase 5 B* (*COX5B*) was significantly downregulated 5.9-fold, and promotor as well as immunocytochemical staining could confirm this effect in silico and on a protein level. The downregulation of COX5B has been reported to decrease cell proliferation and induction of senescence in breast cancer cell lines [62]. The *Tumour necrosis factor receptor superfamily member 14* (*TNFRSF14*) expression was reduced 3.46-fold, and 18 putative NF-κB binding sites were proposed via promotor analysis, which strongly suggests *TNFRSF14* as an NF-κB target. Interestingly, the ligand of the TNFRSF14 receptor, TNFSF14 (LIGHT), has been shown to increase proliferation and cell viability of human bone marrow-derived mesenchymal stem cells [63]. Oppositely, the overexpression of TNFRSF14 has been reported to repress cell proliferation and induce proliferation in bladder cancer [64]. Nevertheless, our results show a reduced protein expression of TNFRSF14 after serum and PDTC treatment in hCSCs, indicating a significant role of TNFRSF14 in proliferation.

In summary, we demonstrate an inductive effect of human blood serum of NF-κB signalling in hCSCs. The inhibition of this pathway via PDTC resulted in reduced proliferation and for increased concentrations in cell death. The simultaneous application of serum and PDTC induced signalling pathways referring to cellular responses to metal ions. In detail, genes encoding for zinc and iron-chelating proteins such as ZNF or MTs were upregulated, indicating a rescue response to PDTC-mediated increased metal ion levels in the cell. We therefore suggest that PDTC mediates cellular Zn^2+^ increases to toxic levels, which cannot be diminished due to inhibited protection of NF-κB. The molecule PDTC does not only possess NF-κB-inhibitory characteristics, but also exhibits metal-ion-related cellular functions. Furthermore, *ATF6B*, *COX5B*, and *TNFRSF14* were identified as putative NF-κB target genes that potentially regulate serum-mediated hCSC proliferation. These results provide an insight into the molecular regulation of the serum-induced proliferation of hCSCs and could contribute to advancements in ageing research and regenerative medicine.

## 4. Materials and Methods

### 4.1. Isolation and Cultivation of Human Cardiac Stem Cells

Human cardiac stem cells were obtained via explant culture from left atrial appendages during routine surgery according to local and international guidelines (Declaration of Helsinki), as described previously [7]. The ethics commission of the Ruhr-University Bochum (Faculty of Medicine, located in Bad Oeynhausen, Germany) conducted ethical approval for isolation and experimental procedures with the approval number eP-2016-148. After surgical removal, biopsies were washed in PBS (Sigma-Aldrich, St. Louis, MO, USA) and cut into small pieces. The tissue clumps were placed on 0.1% gelatin type B (Sigma-Aldrich, Taufkirchen, Germany)-coated TC100 dishes (Sarstedt AG and Co., Nürmbrecht, Germany) and cultivated in human cardiac stem cell medium (hCSC medium) containing DMEM/F-12 (Sigma-Aldrich, St. Louis, MO, USA), basic fibroblast growth factor (bFGF, 5 ng/mL; Peprotech, Hamburg, Germany), epidermal growth factor (EGF, 10 ng/mL; Peprotech), and 10% foetal calf serum (VWR, Radnor, PA, USA). The tissue clumps were removed after reaching confluence, and cells were passaged after detachment with trypsin-EDTA (Sigma-Aldrich, St. Louis, MO, USA). Cells were seeded in gelatin type B-coated T25 cell culture flasks (Sarstedt AG and Co., Nürmbrecht, Germany) for further cultivation. For this study, cells from two female (aged 78 and 80 years) and four male donors (60, 61, 62, and 69 years) were used.

### 4.2. Collection of Human Blood Serum Samples

Human blood plasma samples were collected from six healthy young (age < 20 years) male and six female donors via routine blood donation service, and serum was isolated as described previously [7]. Briefly human blood plasma was obtained via plasmapheresis, and 20% CaCl_2_ in a ratio of 1:50 was added. After incubation overnight, plasma was centrifuged at 1920 RCF for 20 min, and serum was harvested from the supernatant. Serum from six male donors was pooled and serum from six female donors was pooled.

### 4.3. Proliferation Assay

Pre-cultivation of hCSCs was performed under hypoxic conditions with 5% CO_2_ and 5% O_2_ at 37 °C in 0.1% gelatin type B (Sigma-Aldrich, Taufkirchen, Germany)-coated TC25 flasks in hCSC medium containing DMEM-F12 (Life Technologies, Darmstadt, Germany), 10% foetal calf serum, L-glutamine (2 mmol/L), bFGF (5 ng/mL) (Miltenyi Biotech, Bergisch Gladbach, Germany), EGF (10 ng/mL) (Preprotech, Hamburg, Germany), and Penicillin/Streptomycin (10 mg/mL) (Sigma-Aldrich, Taufkirchen, Germany). Cells were detached using Trypsin-EDTA (Sigma-Aldrich, St. Louis, MO, USA) and 1 × 10^3^ hCSCs per well were seeded in a 0.1% gelatin type B-coated 96-TC plate (Sarstedt AG and Co., Nürmbrecht, Germany) in hCSC medium. An assay was performed with two hCSC donors (a 77- and 80-year-old female) and human blood serum from six male donors (age < 20 years) was applied. Technical triplicates were performed for each condition. The medium was changed to starvation medium containing DMEM-F12 (Life Technologies, Darmstadt, Germany), L-glutamine (2 mmol/L), bFGF (5 ng/mL) (Miltenyi Biotech, Bergisch Gladbach, Germany), EGF (10 ng/mL) (Preprotech, Hamburg, Germany), and Penicillin/Streptomycin (10 mg/mL) (Sigma-Aldrich, Taufkirchen, Germany) after 24 h. Subsequent to 72 h of starvation, to calculate the IC_50_ value, 10% pooled blood serum and concentrations from 0 to 750 nM of the NF-κB inhibitor PDTC (Sigma-Aldrich, Taufkirchen, Germany) dissolved in H_2_O were applied. After 72 h of treatment, 10 µL Orangu Cell Counting Solution (Cell Guidance Systems, Cambridge, UK) was added to each well plate for 2 h at 37 °C. Subsequently, absorbance was measured at 450 nm and the cell count was calculated by the formula derived from a previously determined calibration straight. Therefore, hCSCs from the same suspension were seeded in a 0.1% gelatin type B-coated TC-96 well plate with 0, 250, 500, 750, 1000, 1500, 2000, 2500, and 3000 cells per well, and they were allowed to attach to the surface for 1.5 h. Afterwards, 10 µL Orangu Cell Counting Solution (Cell Guidance Systems, Cambridge, UK) was added for 2 h and absorbance was measured at 450 nm.

### 4.4. Immunocytochemical Staining

The cells were cultivated in 8-well slides (ibidi, cells in focus, Gräfelfing, Germany) for immunocytochemical staining in hCSC medium. After 24 h, the medium was changed to serum-deprived medium for 24 h. For the determination of the nuclear translocation of NF-κB subunits, the cells from one male hCSC donor (60 years) were treated with either 10% pooled human blood serum from six male donors (age < 20 years) or 10 ng/mL TNFα for 10 min, 20 min, 30 min, or 40 min, while untreated cells were used as a control. For the determination of the PDTC effects on subunit translocation, hCSCs were treated with pooled blood serum from six male (age < 20 years) or female (age < 20years) donors, or pooled serum with 500 nM PDTC for one hour. Cells receiving treatment with serum and PDTC were incubated beforehand with 500 nM PDTC alone for one hour. For the validation of ATF6B, COX5B, and TNFRSF14, 5 × 10^3^ to 25 × 10^3^ hCSCs from two male donors (age 60 and 61 years) were cultivated in 8-well slides in hCSC medium for 24 h. Subsequently, the medium was changed to serum-deprived medium for another 24 h. Cells were then treated with either 10% pooled serum from six male donors or 10% serum from six male donors with 500 nM PDTC for six hours, while untreated cells served as a control. Cells receiving treatment with serum and PDTC were incubated beforehand with 500 nM PDTC alone for one hour. Afterwards, the medium was discarded, and the cells were washed with PBS followed by fixation at room temperature with 4% PFA for 15 min, followed by three washing steps with PBS. For permeabilization and blocking, 0.02% PBT containing 5% goat serum was applied for 30 min followed by a single wash with PBS. The primary antibodies were applied overnight at 4 °C diluted in a blocking solution. Primary antibodies for RelA (Cell Signalling, Danvers, MA, USA), RelB (Cell Signalling, Danvers, MA, USA), c-Rel (Cell Signalling, Danvers, MA, USA), and TNFRSF14 (Invitrogen, Carlsbad, CA, USA) were diluted at a 1:100 ratio, while primary antibodies for ATF6B (Invitrogen, Carlsbad, CA, USA) and COX5B (Invitrogen, Carlsbad, CA, USA) were diluted at a 1:200 ratio. The second antibody goat anti-rabbit Alexa 555 (Life Technologies, Thermo Fisher Scientific, MA, USA) was diluted in a 1:300 ratio in a blocking solution and applied for one hour at room temperature in the dark. After washing three times with PBS, nuclear counterstaining was conducted by application of 49′,6-diamino-2-phenylindole (DAPI, 1 µg/mL) (Sigma-Aldrich, Taufkirchen, Germany) for 15 min. After washing with PBS two times and one-time H_2_O Mowiol-4-88 was mounted to cover the cells. Fluorescence imaging was performed via confocal laser scanning microscopy (LSM780; Carl Zeiss; Jena, Germany). For analysis, ZEN software (version 2011 SP7) and ImageJ (version 1.54f) were used.

### 4.5. RNA-Sequencing

For RNA-Sequencing, 5 × 10^5^ hCSCs from three male donors (aged 60, 62, and 69 years) were seeded in 0.1% gelatin type B (Sigma-Aldrich, Taufkirchen, Germany)-coated TC100 dishes (Sarstedt AG and Co., Nürmbrecht, Germany) in hCSC medium (described previously). After cultivation for 24 h, the medium was changed to a starvation medium (described previously). After 24 h of starvation, 500 nM PDTC, or the equivalent amount of DMSO, was applied for one hour as a pre-treatment, which was followed by a one-hour treatment with either 10% pooled human blood serum from six young males (age < 20 years) or 10% pooled human blood serum from six young males (age < 20 years) and 500 nM PDTC. RNA was obtained by using the NucleoSpin RNA Kit (Macherey-Nagel GmbH & Co. KG, Düren, Germany), according to the manufacturer’s guidelines, and the amount of isolated RNA was determined using a nanodrop (Peqlab, Erlangen, Germany). The samples were stored at −80 °C. mRNA library preparation and sequencing were conducted by Novogene (Cambridge, UK) with NovaSeq 6000 30 M reads PE150. Reads were aligned to the reference genome GRCh38.p14 using HISAT2 (version 2.1.0-4). To quantify the read number after mapping featureCounts (version 2.0.0) and for differential gene expression analysis, the DESeq2 R package (version 1.42.1) was used. Gene ontology (GO)-term enrichment and Kyoto Encyclopedia of Genes and Genomes (KEGG) pathway analysis was performed by using the gage package in R (version 1.3.1093). For KEGG analysis and GO-Term analysis, the Pathview R package (version 1.3.1) and the org.Hs.eg.db R package (version 3.18) provided databases, respectively.

### 4.6. Promotor Analysis

Promotor analysis was performed in silico according to Slotta et al., 2017 [65] and Kaltschmidt et al., 2018 [39]. Sequences of the relevant promoter regions for Homo sapiens were accessed from the Eukaryotic Promoter Database (https://epd.expasy.org/epd; accessed on 9 September 2023), 2500 bp downstream and 100 bp upstream of the corresponding ATG. The JASPAR Tool [66] was used to identify binding sites for the NF-κB-related transcription factors in the selected promoter sequence, with a relative score threshold of 90%.

### 4.7. Statistical Analysis

For the proliferation assay, significance was determined via a Mann–Whitney U test after the analysis of two hCSC donors, six human blood sera, and after performing technical triplicates. Immunocytochemical investigations were evaluated via Kruskal-Wallis test, and two to five images per condition were analysed for NF-κB subunit translocation and effects of PDTC on serum-induced subunit activation. The investigation of NF-κB subunit translocation in context of time was performed for one hCSC donor, and the application of pooled serum was performed from six male donors. The investigation of the effects of PDTC on serum-induced subunit activation was performed for one hCSC donor as well as for the application of pooled serum from six male donors and pooled serum from six female donors.

## Figures and Tables

**Table 1:** Promotor analysis of downregulated genes after serum and PDTC treatment reveals new putative NF-κB targets. Binding of NF-κB related transcription factors is suggested for the genes *ATF6B*, *COX5B*, and *TNFRSF14*.

Promotor	Name	Score	Start	End	+/−	Predicted Sequence
ATF6B_1	MA0101.1.REL	11.31	1342	1351	+	TGGGGTTTCC
	MA0105.3.NFKB1	12.59	1342	1352	+	TGGGGTTTCCT
	MA0107.1.RELA	11.98	1342	1351	+	TGGGGTTTCC
COX5B_1	MA0105.3.NFKB1	10.71	1499	1509	−	GGGCTTTTCCT
	MA0101.1.REL	9.85	1500	1509	−	GGGCTTTTCC
TNFRSF14_1	MA0105.2.NFKB1	13.66	68	78	−	GGGGGTCTCCC
	MA0105.3.NFKB1	11.37	259	269	+	TGGGGTTCCCT
TNFRSF14_2	MA0105.1.NFKB1	12.80	948	957	+	GGGACATTCC
	MA0101.1.REL	11.13	948	957	+	GGGACATTCC
	MA0105.2.NFKB1	13.67	2224	2234	−	GGGGAGCCCCC
	MA0105.3.NFKB1	12.15	1984	1994	+	GGGCTTTTCCC
	MA0105.3.NFKB1	11.93	1817	1827	−	TGGGCTTCCCT
	MA0101.1.REL	10.33	1070	1079	+	GGGGCTTTTC
	MA0778.1.NFKB2	13.93	2224	2236	+	GGGGGCTCCCCCT
	MA0105.2.NFKB1	13.10	2225	2235	+	GGGGCTCCCCC
	MA0105.3.NFKB1	11.22	1070	1080	+	GGGGCTTTTCC
	MA0778.1.NFKB2	13.45	2224	2236	−	AGGGGGAGCCCCC
TNFRSF14_3	MA0105.1.NFKB1	13.53	1613	1622	−	GGGGCTTCCC
	MA0105.2.NFKB1	14.66	2265	2275	+	GGGGAGTCCCC
	MA0105.3.NFKB1	13.57	1612	1622	-	GGGGCTTCCCA
	MA0105.2.NFKB1	13.66	986	996	−	GGGGGTCTCCC
	MA0105.2.NFKB1	13.13	2265	2275	−	GGGGACTCCCC
	MA0105.1.NFKB1	11.38	2265	2274	+	GGGGAGTCCC
	MA0105.1.NFKB1	11.11	2265	2274	−	GGGACTCCCC

## Data Availability

Data are contained within this article.

## Supplementary Materials

The following supporting information can be downloaded at: https://www.mdpi.com/article/10.3390/ijms25073593/s1.
