# Supplementary material for: Serum Induces the Subunit-Specific Activation of NF-κB in Proliferating Human Cardiac Stem Cells
Source: Int J Mol Sci. 2024 Mar 22;25(7):3593. doi: 10.3390/ijms25073593 (PMC11012129; doi:10.3390/ijms25073593)
Supplement: Supplementary file 1 [file ijms-25-03593-s001.zip › ijms-2896575-supplementary.pdf]

Supplementary Data

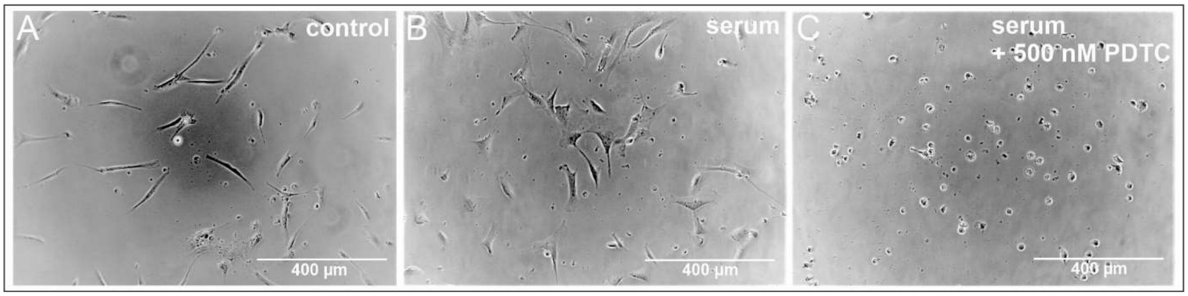

Figure S1: 500 nM PDTC induces cell death of serum-treated hCSCs. Light microscopy images reveal morphological changes indicating cell death after treatment with 500 nM PDTC (A-C).

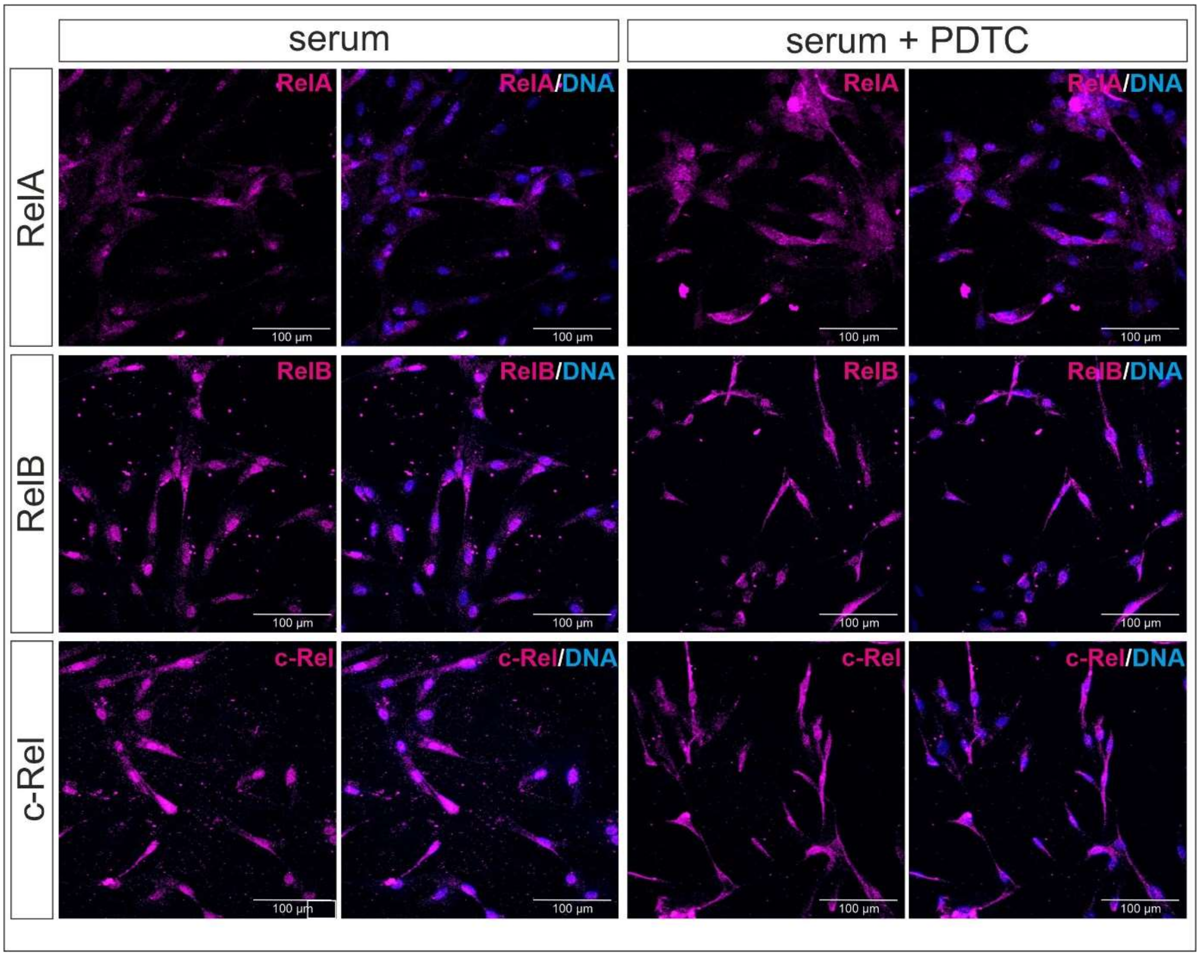

Figure S2: PDTC significantly reduces the nuclear translocation of NF-κB subunits upon serum treatment. The inductive effect of human blood serum on the nuclear translocation of RelA was significantly reduced by PDTC.

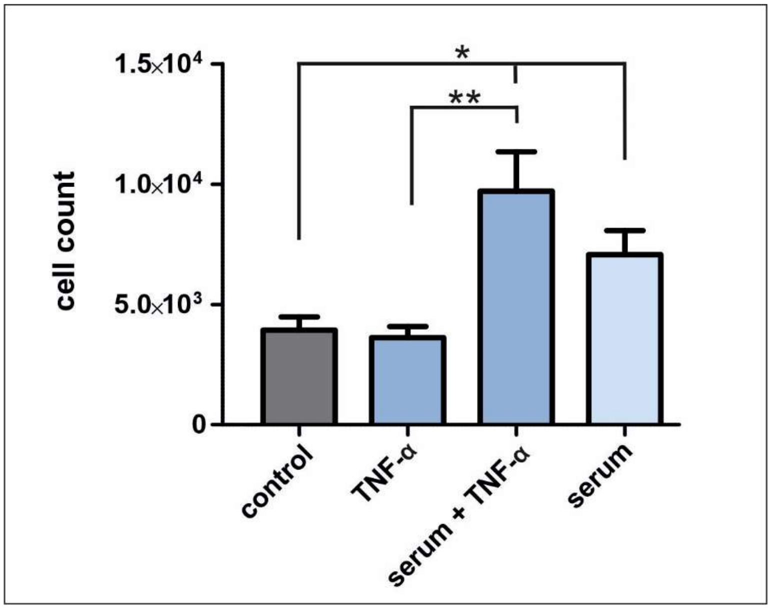

Figure S3: TNFα does not induce hCSC-proliferation. In proliferation assay, after starvation in serum-deprived medium for 72 hours, 10 ng/ml TNFα, 10% human blood serum or serum with 10 ng/ml TNFα were applied to hCSCs for 72 hours. Serum significantly increased proliferation while application of TNFα had no effect on hCSC proliferation. (female hCSC donors n=3, male hCSC donors n=3, human blood serum donors n=6 (pooled); Mann Whitney U, ** $p \leq 0.01$, *$p \leq 0.05$)
